# Advances in the Development of Pharmacological Chaperones for the Mucopolysaccharidoses

**DOI:** 10.3390/ijms21010232

**Published:** 2019-12-29

**Authors:** Juan Camilo Losada Díaz, Jacobo Cepeda del Castillo, Edwin Alexander Rodriguez-López, Carlos J. Alméciga-Díaz

**Affiliations:** 1 Institute for the Study of Inborn Errors of Metabolism, Faculty of Science, Pontificia Universidad Javeriana, Bogotá D.C. 110231, Colombia; juan.losada@javeriana.edu.co (J.C.L.D.); jacobo.cepeda@javeriana.edu.co (J.C.d.C.); rodriguez.edwin@javeriana.edu.co (E.A.R.-L.); 2 Chemistry Department, Faculty of Science, Pontificia Universidad Javeriana, Bogotá D.C. 110231, Colombia

**Keywords:** pharmacological chaperones, lysosomal storage diseases, mucopolysaccharidoses, small molecules

## Abstract

The mucopolysaccharidoses (MPS) are a group of 11 lysosomal storage diseases (LSDs) produced by mutations in the enzymes involved in the lysosomal catabolism of glycosaminoglycans. Most of the mutations affecting these enzymes may lead to changes in processing, folding, glycosylation, pH stability, protein aggregation, and defective transport to the lysosomes. It this sense, it has been proposed that the use of small molecules, called pharmacological chaperones (PCs), can restore the folding, trafficking, and biological activity of mutated enzymes. PCs have the advantages of wide tissue distribution, potential oral administration, lower production cost, and fewer issues of immunogenicity than enzyme replacement therapy. In this paper, we will review the advances in the identification and characterization of PCs for the MPS. These molecules have been described for MPS II, IVA, and IVB, showing a mutation-dependent enhancement of the mutated enzymes. Although the results show the potential of this strategy, further studies should focus in the development of disease-specific cellular models that allow a proper screening and evaluation of PCs. In addition, in vivo evaluation, both pre-clinical and clinical, should be performed, before they can become a real therapeutic strategy for the treatment of MPS patients.

## 1. Introduction

Cells and organisms are particularly sensible to alterations in their homeostasis and proteostasis, the latter meaning the appropriate production, transport, folding, turnover, function, and disposal of proteins [1]. In cellular stress conditions, such as overproduction of misfolded proteins, temperature increases, or oxidative stress, both heat shock response and unfolded protein response (UPR) can be activated [2]. Additionally, the expression of a certain type of proteins called chaperones is increased. These proteins, also called molecular chaperones, act on proteins that reach the endoplasmic reticulum (ER) and on some cytosolic proteins, aiding in the proper folding of unfolded and misfolded proteins. In this sense, molecular chaperones reduce the amount of proteins that are targeted for degradation by decreasing their aggregation and proteasome targeting [3]. The ER environment is very different from that of the cytosol since it has an oxidizing nature that helps the disulfide bond formation and it also has a mM concentration of Ca^2+^, which acts as a folding buffer [4]. Most molecular chaperones act by shielding the hydrophobic residues exposed in the misfolded proteins, especially in soluble proteins, and directing them towards the inner part of the protein conformation [1]. However, in some cases, misfolded proteins build up in the ER leading to ER stress and a steady UPR activation, which may lead to a high production of reactive oxygen species and cell death [5]. The ubiquitin proteasome degradation system (UPS) is key in the clearance of soluble misfolded proteins in association with the ER-associated degradation (ERAD), but the UPS fails to act on insoluble protein aggregates often causing proteotoxicity [6], which can be reduced by the action of molecular chaperones [7].

Protein folding itself starts during synthesis, and co-translational and post-translational folding are continuous processes. In some cases, the disulfide bond formation can be completed even after translation or after translocation of the proteins [8]. Some of the main chaperones that have been characterized use cycles of ATP binding and hydrolysis as they act on newly synthesized polypeptides, while others simply protect and shield proteins during their translation [9]. Two of the most studied ATP binding molecular chaperones are the heat shock response proteins HSP70 and HSP90. HSP70 can prevent misfolding and aggregation or keep the proteins unfolded until their transport to their final cellular destination. In eukaryotes, HSP70 can also associate with the RAC protein and appears to couple co-translational folding with peptide elongation by the ribosome [10,11]. HSP70 acts by stabilizing the unfolded or misfolded proteins, which, upon release from the chaperone, can spontaneously adopt their correct folding. On the other hand, HSP90 seems to bind unfolded and misfolded proteins at late stages of the post-translational modification and promote their correct folding, thus preventing their aggregation [9]. Some of the proteins that fail to gain a correct folding by the action of ribosome-associated chaperones are folded in the ER aided by the HSP70 system. This system, in addition to preventing unproductive interdomain interactions and promoting cotranslational folding, provides connections to other downstream chaperones [12].

The concept of molecular chaperones and how they aid in the rescuing and correct folding of proteins extends to small molecules that can participate in the protein folding process. These molecules, called pharmacological chaperones (PCs), bind to target proteins with high specificity, stabilizing the native conformation or promoting the correct folding of misfolded and unfolded proteins [13,14]. On the ER lumen, PCs bind to proteins of the secretory pathway and facilitate the correct folding and export out of the organelle, reducing the amount of protein that is aggregated in the ER or targeted to the proteasome [3]. The chaperoning potency has been linked to the binding affinity of the PC, and the most energetically favorable interactions are those that elicit the best folding responses [15]. A problem that may arise in the treatment of diseases that involve mutant and misfolded proteins, such as many of the lysosomal storage diseases (LSDs), is that the PC could bind with too high affinity to the orthosteric site of the protein (i.e., the active cavity), and compete with the natural substrate of the protein (i.e., inhibitory PC), thus preventing its proper activity [16,17]. Nevertheless, PCs could be dissociated from the protein in response to the acidic lysosome environment making the active site available once again. In the case of competitive inhibition, the problem could be solved by targeting allosteric sites of the protein (i.e., non-inhibitory PCs).

One of the relevant uses of PCs is as a treatment alternative to LSDs, since some of the mutations causing these diseases can cause mutated enzymes to present conformational alterations that prevent the enzymes from passing the cells’ quality control mechanisms, resulting in substrate build-up and corresponding pathologies. In this context, PCs can stabilize the mutant enzymes, aid in their proper folding, and promote their correct trafficking to the lysosome [18]. It has been proposed that, compared to enzyme replacement therapy (ERT), PCs have advantages of oral administration, wide tissue distribution profile, and fewer issues of immunogenicity [19,20]. In addition, PCs have also been studied in combination with ERT, and it seems that PCs, aside from acting on the patient mutant enzymes, can act on the recombinant enzyme as well, increasing the stability, delivery, and half-life of the enzyme [21]. On the other hand, the main disadvantage of this therapeutic strategy is that not all mutations may be responsive to the PC.

One of the approaches to identify PCs is to test the chaperoning potential of molecules that resemble the natural substrate structure (glycomimetics), as in the case of testing imino-sugars as orthosteric PC for the mutated β-hexosaminidase A (HexA) enzyme in Tay–Sachs disease [22]. Nevertheless, non-substrate-like PCs have been described in Gaucher [23,24] and Krabbe [25] diseases. Another approach is to test in vitro the chaperoning potential of large sets of molecules, which can be done for example through differential scanning fluorimetry (DSF). This method measures a protein resistance to thermal denaturation in the presence of potential PCs using a fluorescent dye that increases the signaling when bound to unfolded or misfolded proteins [26]. PCs can also be tested using cell-based screens as done by the identification of PCs for the vasopressin type 2 receptor [27]. To avoid the high costs of an in vitro screening, virtual screening can also be used, especially if there is a crystal structure of the protein under evaluation. These screenings are done performing in silico simulations of molecular dockings of a molecule database with the protein, thus allowing the identification of those molecules that have the best affinities for the protein orthosteric or allosteric sites [28,29]. In this paper, we will review the recent advances of PC for LSDs with a special emphasis in those small molecules identified as PCs for the mucopolysaccharidoses (MPS).

## 2. Use of Pharmacological Chaperones in LSDs

LSDs are a group of about 50 inborn errors of metabolism (IEM) characterized by the lysosomal accumulation of partially or non-degraded molecules. Therapies for LSDs treatment may include: (1) to promote the storage clearance by administering an exogenous functional enzyme, (i.e., ERT); (2) to prevent the lysosomal accumulation of substrates by inhibiting their synthesis (i.e., substrate reduction therapy); (3) to enhance the enzyme activity of a mutated protein (i.e., PC therapy); and (4) to perform a bone marrow or stem cell transplantation, especially in those diseases with central nervous system (CNS) involvement [30,31,32,33,34,35,36]. Molecular characterization of LSDs has shown that in most of the cases the disease is caused by missense mutations [37,38]. Pathophysiological studies in different LSDs have suggested that in some cases, the amino acid change leads to loss of protein stability due to changes in their processing, folding, glycosylation, and pH stability, which promotes aggregation and can induce ER or Golgi apparatus retention and/or increased degradation, or defective transport to the lysosomes [37,38,39,40,41,42,43,44,45,46,47,48]. In this sense, the use of PCs as an alternative to restore the folding and trafficking of the mutated lysosomal proteins has been proposed [19,20]. It has been observed that PCs can increase the enzyme activity of the mutant proteins in a mutation-dependent manner, which may be sufficient to ameliorate most disease symptoms [49,50]. In addition, it has been observed that these drugs can enhance the stability of recombinant lysosomal enzymes, suggesting that co-administration of a PC and recombinant enzyme may increase the efficacy of the ERT [3,51]. Here, we will highlight some of the studies that have identified and characterized PCs for LSDs.

The first promising results of a PC for LSD treatment were observed around 20 years ago when it was demonstrated that treating cells from Fabry patients with an α-galactose (α-GAL) analogue induced an improvement of the lysosomal trafficking of the enzyme and increased the enzyme activity [52,53]. In fact, the PC therapy for Fabry disease by using 1-deoxygalactonojirimycin (DGJ, migalastat) gained FDA approval in 2016 [54]. Schindler–Kanzaki disease (Schindler disease type II) is a very rare lysosomal storage disorder characterized by the deficiency in the activity of the α-N-acetylgalactosaminidase enzyme (α-NAGAL). It was reported that the DGJ can inhibit α-NAGAL in similar form to α-GAL in Fabry disease. Likewise, a related iminosugar, 2-acetamido-1,2-dideoxy-d-galactonojirimycin (DGJNAc), was synthesized and tested, showing an improved specificity for α-NAGAL. Both DGJ and DGJNAc were able to protect human α-NAGAL from proteolytic degradation and increased the amount of wild-type α-NAGAL produced by HEK 293T cells [55].

PCs have been also extensively evaluated for the treatment of Gaucher disease type 2 (GD2) and type 3 (GD3) [56]. Initially, GD patients were treated with isofagomine tartrate (a reversible competitive inhibitor of glucocerebrosidase, GCase). However, the results obtained in a phase II clinical trial did not show a significant effect in the clinical manifestations of the disease [56]. In a non-randomized study including five GD patients, ambroxol combined with ERT significantly increased GCase activity in lymphocytes, and positively impacted patients with myoclonus, seizures, and pupillary light reflex dysfunction [57]. Recently, a study assessed the effect of oral ambroxol supplementation in combination with ERT and an improvement in glucosylsphingosine levels on dried blood spots samples from GD2 and GD3 patients was observed. However, a variable clinical response was observed in the ambroxol treated patients, suggesting a genotype-dependent chaperone effect of this PC [58]. Non-inhibitory chaperones (i.e., allosteric binding molecules) have also been described as potential PCs for GD, since they can increase GCase activity, reduce substrate accumulation, and restore chemotaxis [59]. These non-inhibitory PCs have also shown promising results, allowing a reduction of α-synuclein levels in induced pluripotent stem cell-derived dopaminergic neurons [60]. However, the identification of potential non-inhibitory PC requires the development of specific substrates that can measure the GCase activity within the lysosome [61].

The use of azasugar 4-*epi*-isofagomine (4-*epi*-IFG) as a PC has also been described as possible therapy for Krabbe disease. This compound showed a 98% inhibition of β-galactocerebrosidase (GALC) with a half maximal inhibitory concentration (IC_50_) of 1 µM. However, this molecule lacks selectivity, as it was also able to inhibit lysosomal β-galactosidase [62,63]. N-ocytl-4-epi-b-valienamine (NOEV) was tested in cells from Krabbe disease patients, but the use of this compound as PC for Krabbe disease treatment remains unclear [63,64]. On the other hand, the alkaloid α-lobeline was identified as an allosteric PC for GALC. This molecule increased the enzyme’s activity on a neuronal cell line with the GALC mutation p.D528N, while no effect was observed in other tested mutations [63,65]. Similar results were observed in fibroblasts from Krabbe disease patients treated with the allosteric PC α-lobeline and 3′4′7-trihydroxyisoflavone [25,63].

Pompe disease is an LSD caused by mutations of the gene that encodes for the enzyme acid α-glucosidase (GAA). N-acetylcysteine (NAC), a compound that was proposed as an allosteric chaperone for GAA, showed promising results in vitro [66]. This PC enhanced the enzymatic activity of a recombinant GAA, improving the efficacy of ERT in Pompe disease [66,67]. Additionally, NAC enhanced the activity of mutated GAA in Pompe fibroblasts and in COS7 cells overexpressing mutated GAA [66]. Similarly, N-butyl-deoxynojirimycin (NB-DNJ) showed a more efficient correction of enzyme activity of GAA in combination with recombinant GAA in Pompe fibroblasts. Overall, NB-DNJ improved the trafficking of mutant GAA to lysosomes, enhanced the enzyme maturation, and increased the enzyme stability in Pompe disease fibroblasts and in a Pompe mouse model [67,68]. These results led to the design of a clinical trial to evaluate the effect of a combined ERT and PC treatment strategy, showing a significant increase in blood GAA activity, suggesting an improved stability of recombinant GAA in blood in the presence of the PC [69].

In galactosialidosis, caused by a combined deficiency of β-galactosidase and N-acetyl-α-neuraminidase due to a primary defect of protective protein/cathepsin A, it was observed that NOEV can enhance β-galactosidase activity in skin fibroblasts from patients with the early infantile and the juvenile/adult form [67,70]. Similarly, pyrimethamine (PYR) was identified as a competitive inhibitor of β-hexosaminidase, thus representing an alternative for the treatment of Tay–Sachs and Sandhoff diseases, since it produced an increase in the enzyme activity in 44% of the evaluated mutations [71]. The use of PYR reached phase II clinical trials for treatment of patients affected with late-onset GM2 gangliosidosis. A total of eight patients were evaluated during 16 weeks at doses of up to 50 mg/day, reaching a 4-fold enhancement of HexA activity in leukocytes [67,72]. On aspartylglucosaminuria, an LSD caused by mutations in the gene coding for aspartylglucosaminidase (AGA), glycine and aspartic acid have been tested as PC. Although these compounds increased the AGA activity in aspartylglucosaminuria fibroblasts, their use in therapy might be limited due to significantly inhibited AGA enzyme activity [73]. Betaine (trimethylglicine), has been also tested as a PC of AGA, which seems to improve the metabolic/osmotic state of the cells, relieving ER and lysosomal stress and producing an improvement in AGA folding and activity [73].

Finally, the possibility of designing pharmacological chaperons based on the 5N,6O-oxomethylidenemannojirimycin-β-cyclodextrin conjugate (OMJ−βCD) for the treatment of α-mannosidosis has been proposed, an LSD that is caused by a deficient α-mannosidase (LAMAN) activity. Monovalent OMJ derivatives equipped with aglycone moieties significantly enhanced mutant LAMAN activity in fibroblasts from patients and in MAN2B1-KO HAP1 cells expressing several LAMAN mutations. The OMJ−βCD conjugate exhibited high efficiency in folding-defective mutants at very low (2 nM) concentrations. Another OMJ compound greatly enhanced the activity of the active site H72L/H72L mutant by increasing its overall stability. This response was accompanied by an increase in the protein traffic from the ER/Golgi to the lysosome and by a reduction of cholesterol content in the cell [74].

## 3. Pharmacological Chaperones for MPS

MPS are genetic disorders caused by the deficiency of 1 of 11 lysosomal enzymes involved in the metabolism of glycosaminoglycans (GAGs). Enzyme deficiencies may lead to the accumulation of the GAGs heparan sulfate (HS), dermatan sulfate (DS), keratan sulfate (KS), chondroitin sulfate (CS), or hyaluronan within the lysosomes [75]. As a result, partially catabolized GAGs accumulate in the lysosomes of several tissues and are secreted into the blood and excreted in urine [76]. There are seven types of MPS that are categorized based on the lysosomal enzyme that is deficient (Table 1). Treatment options for MPSs may include ERT or hematopoietic stem cell therapy (HSCT) and other options such as gene therapy, genome editing, and PCs are being studied [77]. In this section, we will summarize the in vitro and in vivo studies reporting the identification and characterization of PC for MPS.

### 3.1. Mucopolysaccharidosis Type II

Mucopolysaccharidosis type II (MPS II, Hunter syndrome, OMIM 309900) is an X-linked disease caused by the deficiency of iduronate-2-sulafatase (IDS). This enzyme deficiency leads to the progressive lysosomal accumulation of the HS and DS, presenting systemic manifestations such as skeletal deformities, mental retardation, valvular heart disease, hepatosplenomegaly, and skin abnormalities [78].

Currently, ERT with recombinant human IDS (idursulfase and idursulfase-beta) is the standard treatment for MPS II patients. These ERTs have shown to be safe therapies for MPS II patients, and are effective in relation to functional capacity, urinary GAGs, and liver and spleen volume for attenuate and severe phenotypes. Nevertheless, limited effects have been observed in growth and cognitive disease [77,79]. HSCT has not been recommended for MPS II patients due to the lack of CNS improvement of this treatment strategy [78].

**Figure 1:**
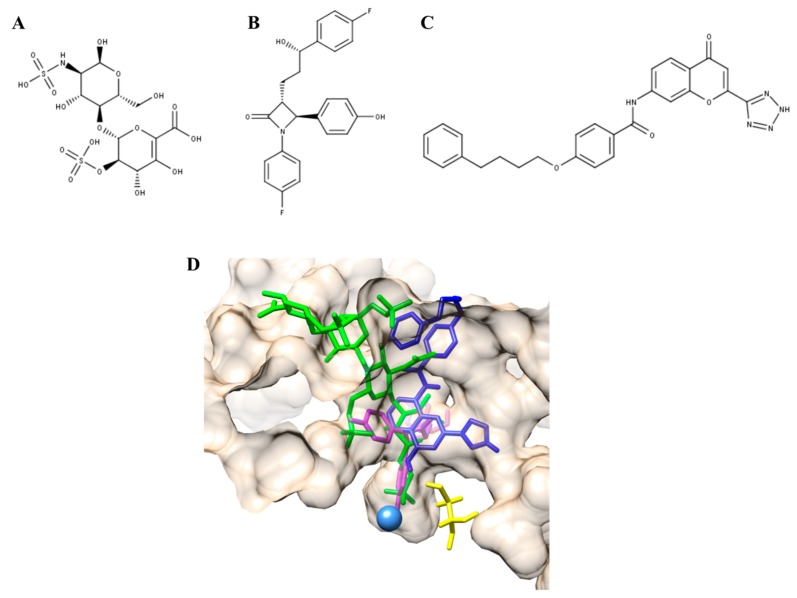
Pharmacological chaperones (PCs) for MPS II and MPS IVA. (**A**) Δ-unsaturated 2-sulfouronic acid-N-sulfoglucosamine (D2S0), (**B**) ezetimibe, and (**C**) pranlukast have been described as PC for (**A**) MPS II and (**B**,**C**) MPS IVA. (**D**) PC for MPS IVA were predicted to stablish similar interactions to those predicted for the natural and artificial N-acetylgalactosamine-6-sulfate sulfatase (GALNS) substrates. Green, KS; magenta, ezetimibe; blue, pranlukast. The catalytic residue (C79) and calcium ion are colored in yellow and light blue, respectively.

Recently, a PC was described as a potential treatment alternative for MPS II patients [78]. In this study, the authors described the use of Δ-unsaturated 2-sulfouronic acid-N-sulfoglucosamine (D2S0, Figure 1A), which is a sulfated disaccharide derived from heparin. D2S0 has a similar structure to 2-O-sulfated iduronic acid-linked N-sulfated glucosamine, which is a natural substrate of IDS. The results showed that D2S0 has an IC_50_ of 30.1 µM and competes with the 4-methylumbelliferyl IDS artificial substrate, suggesting that this compound interacts with the active cavity of IDS. Recombinant human IDS (rhIDS) activity was significantly reduced (by about 25%) after incubation at 67 °C during 1 h; while the incubation with D2S0 increased, by about 5%, the thermal stability of rhIDS at this temperature, and in a dose-dependent manner. In MPS II fibroblasts, carrying the p.231L mutation, the treatment with 10 µM D2S0 allowed a 1.97-fold increase in IDS activity. The analysis of pathological iduronic acid from non-reducing end of GAGs did not show a significant change after 4 days post-treatment with D2S0; while a significant reduction in Alcian blue-positive granules (60%) was observed after 8 days post-treatment with D2S0. These results show the therapeutic potential of D2S0 and suggest that long D2S0 treatment could be necessary to reduce GAGs accumulation. In addition, the treatment of HEK293T cells expressing IDS mutants p.N63D, p.L67P, p.A85T, p.R88H, p.Y108S, and p.P231L with 10 µM D2S0 led to an increase in IDS activity between 1.6- and 39.6-fold, while no effect was observed in cells expressing p.L314P IDS. On the other hand, the p.A85T mutation showed a 7.7-fold reduction in IDS activity at 10 µM; at 0.1 µM a 1.7-fold increase was observed. In this sense, these results showed that D2S0 may function as a PC for IDS in a mutation-dependent manner. Nevertheless, enzyme activity values after treatment with D2S0 were between 2.0% and 7.0% of those observed for wild-type IDS, suggesting that further drug optimization through medicinal chemistry could be necessary to increase the efficacy of this molecule. However, this strategy could represent an interesting alternative for MPS II since about 41% of mutations affecting the *IDS* gene are missense. In addition, about 90% of these missense mutations are involved in amino acids that are buried within the fold of IDS, and it is expected that these mutations can induce local destabilization and misfolding, leading to premature degradation [80].

### 3.2. Mucopolysaccharidosis Type IVA

Mucopolysaccharidosis type IVA (MPS IVA, Morquio A syndrome, OMIM 252300) is an LSD caused by mutations in the gene encoding for the lysosomal enzyme N-acetylgalactosamine-6-sulfate sulfatase (GALNS) [81]. This enzyme deficiency leads to the lysosomal accumulation of the KS and chondroitin-6-sulfate (C6S) [81,82]. Clinically, MPS IVA patients are characterized by short stature, corneal clouding, hypoplasia of the odontoid process, pectus carinatum, valvular heart disease, mild hepatomegaly, laxity of joints, kyphoscoliosis, and genu valgum without CNS impairment [81,82,83]. Treatment options for MPS IVA patients include non-steroidal anti-inflammatory drugs, antibiotics, oxygen supplementation, surgical procedures to correct orthopedic deformities and tracheal obstructions, ERT, and HSCT [81,82,84]. ERT has showed slight improvement in the 6-min walk test and the reduction of urinary KS [85], as well as an improvement in the maximal voluntary ventilation and performance of daily life activities [86]. Unfortunately, ERT has limited effects in correcting the abnormal growth and the skeletal, corneal, and cardiac abnormalities, due to tissue avascularity and short half-life of the enzyme [87,88,89]. On the other hand, although HSCT treatment showed long-term and normal enzyme activities, increase in lumbar bone mineral density, improvement in ambulatory movement and remission of the narrow airway [90,91,92], this is a high-risk procedure with many possible complications and high mortality. In this sense, it is necessary to explore new therapeutic strategies for MPS IVA to improve and expand the treatment options for MPS IVA patients.

Recently, the identification and characterization of the first PCs for MPS IVA was described [93]. Based on results from a modeled GALNS and computational dockings for natural and artificial substrates, a virtual screening strategy against 11,421 compounds tested in humans (ZINC In Man, a special subset of ZINC [94]), was designed to identify a set of compounds that bind to the active cavity of the enzyme. Ezetimibe and pranlukast (Figure 1B,C) were selected among the top 20 interacting compounds and were predicted to establish similar interactions to those modeled for KS and C6S, and the artificial GALNS substrate [48,93] (Figure 1D). Ezetimibe is an approved drug that blocks intestinal cholesterol absorption by selectively inhibiting Niemann-Pick C1-like 1 protein and is indicated for treatment of disorders with elevated cholesterol levels [48]. Pranlukast is an orally administered, selective, and competitive cysteinyl leukotriene type 1 receptor antagonist, indicated for the treatment of bronchial asthma in pediatric and adult patients [95]. In terms of safety, both drugs are well tolerated and have adverse event profiles similar to placebo [48,95].

In vitro evaluation showed that ezetimibe and pranlukast bind to the active cavity of the enzyme since they competed with the 4-methylumbelliferyl substrate (between 40% and 50% inhibition) and increased the thermal stability of the enzyme (5 °C). It was also observed that both compounds significantly increase the activity of human recombinant GALNS (hrGALNS) produced in bacteria (*Escherichia coli*), yeasts (*Pichia pastoris*), and mammalian cells (HEK293), with activities between 1.3- and 5.0-fold higher than in control cultures. This is an important observation, since protein folding represents a bottleneck in the production of recombinant proteins, reducing not only the protein’s biological activity, but also triggering cellular stress, which results in low production yields [96,97]. In this sense, these results show a potential application of ezetimibe and pranlukast to improve production yield and activity of hrGALNS, which may help to reduce immunogenicity of ERT, as the amount of protein used in ERT can be reduced [98].

Finally, both compounds were assayed in MPS IVA fibroblasts carrying the p.R61W, p.R94C, p.F285del, p.A393S, and p.W405_T406del mutations. In the case of ezetimibe, GALNS activity was increased in all treated cells between 1.4- and 2.5-fold compared to untreated cells, reaching, in some cases, similar levels to those observed in wild-type cells. On the other hand, pranlukast increased GALNS activity fibroblasts with p.R61W/p.W405_T406del and p.A393S mutations, albeit less effectively than ezetimibe. The results showed that the lower the concentration of the PC was, the greater the increase in the activity was, with the best results observed at 0.001 µM for both PCs. In addition, treatment of the MPS IVA fibroblasts with these PCs showed an increase in GALNS protein, reduction of lysosomal mass, and normalization of the autophagy markers LC3B and p62. Of note, it was observed that the combination therapy of hrGALNS with ezetimibe or pranlukast additively reduced lysosomal mass in patient-derived fibroblasts. In this sense, this study showed that ezetimibe and pranlukast are PCs that have the potential for use as a monotherapy for MPS IVA patients and could be also considered in the design of a combination therapy with ERT, which may improve the safety and efficacy of ERT for MPS IVA patients [93].

### 3.3. Mucopolysaccharidosis Type IVB

**Figure 2:**
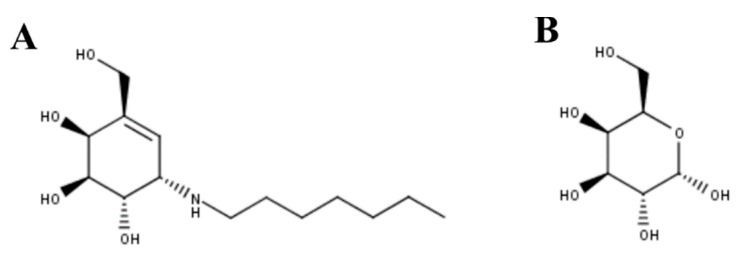
(**A**) N-octyl-4-epi-β-valienamine (NOEV) and (**B**) galactose were the first PCs described for GLB1 deficiency.

**Figure 3:**
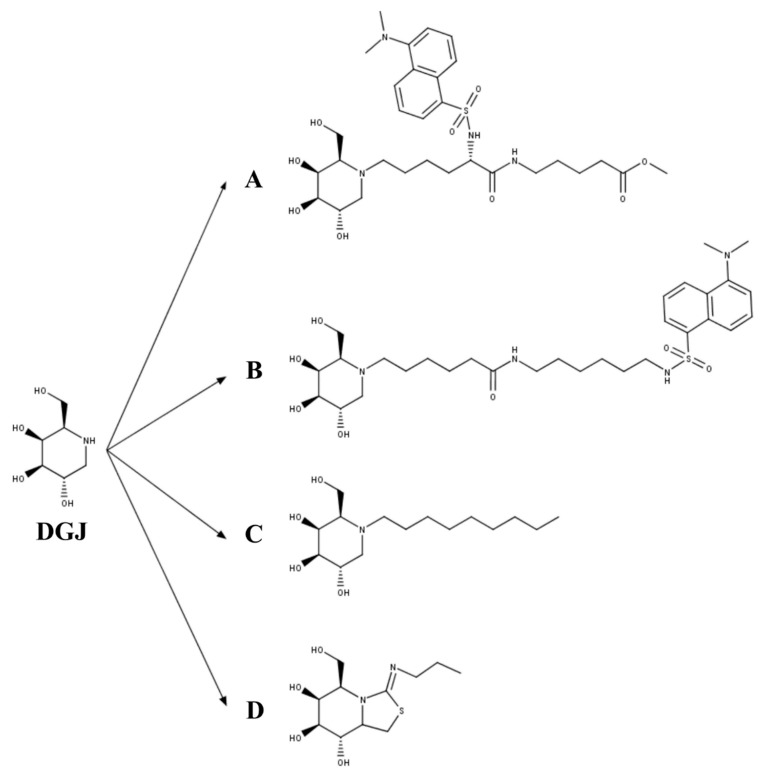
The 1-deoxygalactonojirimycin (DGJ)-derived PCs for GLB1 deficiency. (**A)** Methyl 6-{[N2-(dansyl)-N6-(1,5-dideoxy-d-galactitol-1,5-diyl)-L-lysyl]amino} hexanoate (DLHex-DGJ); (**B**) N-(dansylamino) hexylaminocarbonylpentyl-1,5-dideoxy-1,5-imino-d-galactitol; (**C**) N-nonyl-deoxygalactonojirimycin (NN-DGJ); and (**D**) 5N,6S-(N-butyliminomethylidene)-6-thio-1-deoxygalactonojirimycin (6S-NBI-DGJ).

**Figure 4:**
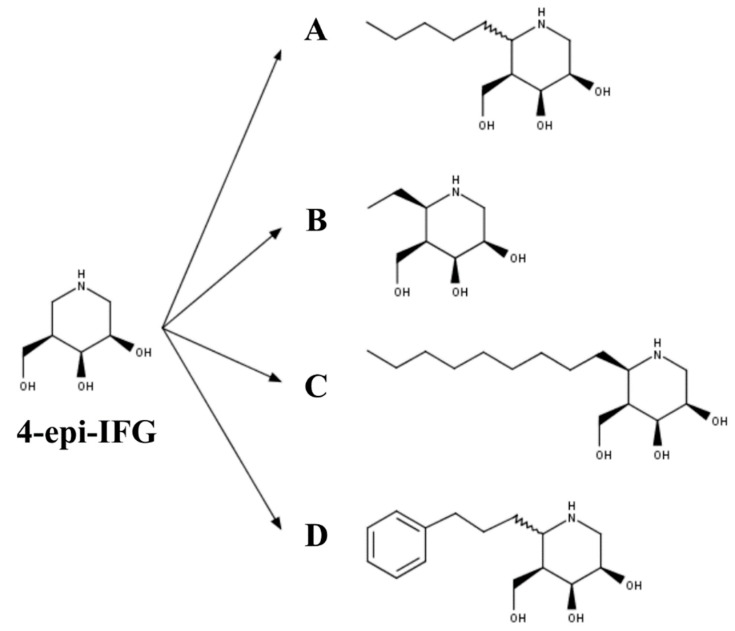
The 4-*epi*-isofagomine (4-*epi*-IFG) derived PC for GLB1 deficiency: (**A**) (5aS)- and (5aR)-5a-C-pentyl-4-*epi*-IFG; (**B**) 5a-C-methyl-4-*epi*-IFG; (**C**) 5a-C-nonyl-4-*epi*-IFG; and (**D**) 5a-C-2-phenylethyl-4-*epi*-IFG.

Deficiency of β-galactosidase (GLB1) causes two clinically distinct phenotypes: GM1-gangliosidosis (OMIM 230500) and mucopolysaccharidosis type IVB (MPS IVB, Morquio B syndrome, OMIM 253010). GLB1 deficiency leads to the impairment of the metabolism of GM1-gangliosides, glycoproteins, oligosaccharides, and KS, which accumulate in tissues and urine from patients with these clinical phenotypes [99,100]. GM1 gangliosidosis includes phenotypes that range from severe to mild, with a progressive CNS dysfunction that may include spasticity, deafness, blindness, decerebrate rigidity, insidious plateauing of motor and cognitive development followed by slow regression, gait disturbance, and cardiomyopathy. In some cases, skeletal involvement could be present including short stature, kyphosis, and scoliosis [101]. MPS IVB is characterized by generalized skeletal dysplasia including short-truck dwarfism, genu valgus, kyphosis, and short neck; as well as extra-skeletal manifestations such as corneal deposits, hepatomegaly, small teeth, and cardiac valvular lesions [100]. Currently, 185 mutations have been reported in GLB1, and about 70% of them are missense mutations. No specific treatment is available for these diseases and the patients are usually treated through symptomatic therapies [99,100]. In contrast to MPS II and MPS IVA, several PCs have been described for β-galactosidase deficiencies (Figure 2, Figure 3 and Figure 4 and Table 2). Although most of these studies have been performed for GM1-gangliosidosis-associated mutations, the results could be extrapolated to MPS IVB since the genetic defect affects the same enzyme.

NOEV was the first PC described for GLB1 deficiency [102]. NOEV (Figure 2A) has an IC_50_ of 0.2 µM against human GLB1 and increased the enzyme activity between 1.2- and 5.1-fold in mouse fibroblasts expressing GLB1 carrying GM1-gangliosidosis (p.R201C and p.R201H/p.R457Q) or MPS IVB (p.W273L/p.Y83H) mutations. Similar results were observed in human fibroblasts from GM1-gangliosidosis patients. In vivo evaluation of NOEV was performed in a mouse expressing the human p.R201C-mutant GLB1 but lacking the endogenous mouse β-galactosidase. Oral administration of NOEV (1 mM ad libitum for 1 week, which corresponds approximately to 1.4 mg per day) led to a significant increase of GLB1 activity in cerebrum, cerebellum, heart, lung, liver, spleen, kidney, muscle, and plasma; with heart, lung, spleen, and muscle showing the greatest improvements. As observed by an immunostaining analysis of the brain, GM1 and GA1 storage was reduced in the frontotemporal cerebral cortex and brainstem, which is a surprising result due to the short term of the treatment. In fact, mass biochemical analysis of brains from treated mice (i.e., lipids analysis by TLC) did not show reduction in GM1 and GA1. In this sense, although the results showed the potential of NOEV as a PC for GLB1 deficiencies, long-term studies should be performed to establish the optimal dose to evaluate the effect of this PC on substrate reduction, and to observe the potential adverse effects.

Galactose was then evaluated as potential chemical chaperone for GLB1 (Figure 2B) [103]. Galactose competitively bound to the active cavity of β-galactosidase, since a significant reduction in enzyme activity (92%) was observed in wild-type human fibroblasts treated with 200 mM galactose. Treatment of GM1-gangliosidosis patient fibroblasts with 200 mM galactose showed a 2.5-fold increase in enzyme activity in cells carrying the p.T329A/p.R442Q mutations; while no effect was observed in fibroblasts carrying the p.S54N/p.R59C and p.R201H/p.G579D mutations. Similarly, the addition of 200 mM galactose to COS-1 cells expressing a p.R442Q mutated β-galactosidase showed a 1.7-fold increase in enzyme activity. These results show the potential of galactose as a PC for GLB1 deficiency.

A third set of PCs for GLB1 have been derived from DGJ (Figure 3), a well-known competitive inhibitor of lysosomal glycosidases that is approved for the treatment of Fabry disease [54]. Fantur et al. [104] evaluated the compound methyl 6-{[N2-(dansyl)-N6-(1,5-dideoxy-d-galactitol-1,5-diyl)-L-lysyl]amino} hexanoate (DLHex-DGJ) as a PC for GLB1 (Figure 3A). It was expected that the chemical modifications done on the DGJ core to produce DLHex-DGJ increased the lipophilicity and the inhibition activity compared to DGJ. DLHex-DGJ showed a *K_i_* of 0.6 µM and an IC_50_ of 6 µM; it was 10-fold more effective than DGJ, resulting in approximately 94% inhibition of a human wild-type GLB1. The treatment of GM1-gangliosidosis and MPS IVB patient fibroblasts with 20 to 500 µM DLHex-DGJ showed a 1.3- to 12.5-fold increase in GLB1 activity depending on the mutation, reaching values between 0.4% and 57.2% of those from wild-type fibroblasts (Table 2). These results showed that although DLHex-DGJ increased the activity of all tested mutations, this increment could not be enough to have clinical impact on the disease phenotype. Of note, the highest increase in enzyme activity was observed on fibroblasts carrying mutations at p.R201 (p.R201C and p.R201H), which were associated with production of misfolded and unstable precursor proteins rapidly degraded by ERAD. In these mutations, treatment with DLHex-DGJ enhanced the amount of mature enzyme and restored the intracellular trafficking of the enzyme. Similarly, Fröhlich et al. [105] evaluated the chaperone capacity of the N-(dansylamino)hexylaminocarbonylpentyl-1,5-dideoxy-1,5-imino-d-galactitol (Figure 3B). This compound showed a *K_i_* of 0.7 µM in human GLB1, and a greater chaperone effect than DLHex-DGJ, since significant increase in enzyme activity was observed even at 1 µM (approximately 2-fold increase) reaching up to a 10-fold increase after 500 µM treatment of human fibroblasts with the p.R201C mutation. Nevertheless, the results showed that N-(dansylamino) hexylaminocarbonylpentyl-1,5-dideoxy-1,5-imino-d-galactitol was less effective than NOEV. A third DGJ-derived PC evaluated for GLB1 deficiency was N-nonyl-deoxygalactonojirimycin (NN-DGJ, Figure 3C) [106]. This compound is a competitive inhibitor of human GLB1 and has an IC_50_ of 0.04 µM at pH 6.5 and of 0.120 µM at pH 4.3, thus suggesting a lower affinity at the lysosome than in the ER. NN-DGJ also increased the thermostability of wild-type human GLB1 between 3- and 4-fold, confirming the direct interaction of NN-DGJ with the enzyme and the chaperone activity of NN-DGJ. Evaluation in GM1 fibroblasts carrying the mutations p.R148S/p.D332N showed that a significant increase in GLB1 activity was only observed with concentrations 15-fold greater than that of the IC_50_, reaching a 2- to 3-fold increase in activity with concentrations between 1.0 and 2.6 µM. Treatment of GM1 fibroblasts with 1.2 µM NN-DGJ restored the intracellular trafficking of the mutated enzyme, reducing the GLB1 present in the ER and increasing the amount of GLB1 in the lysosome. Evaluation on GM1 and MPS IVB human fibroblasts showed 5- and 7-fold increases in enzyme activity on fibroblasts with mutations p.R201H/IVS14-2A>G and p.R201H/p.W509C, respectively, favoring also a correct maturation of these mutated enzymes. However, other GM1 and MPS IVB fibroblasts were not responsive to the treatment with NN-DGJ. Finally, a bicyclic DGJ derivative was evaluated as a novel PC for GLB1 [107]. This derivative, namely 5N,6S-(N-butyliminomethylidene)-6-thio-1-deoxygalactonojirimycin (6S-NBI-DGJ, Figure 3D), inhibits wild-type human GLB1 with an IC_50_ of 32 µM, and significantly increases the thermostability of the enzyme. Treatment of GM1 patient fibroblasts with 20 and 80 µM 6S-NBI-DGJ showed a significant improvement of GLB1 activity (between 2- and 3-fold) for those fibroblasts carrying the mutations p.I51T, p.I51T/p.Y316C, p.I51T/p.R457Q, p.G190D, p.R201C, p.G438E, and p.R457Q; no response was observed for p.R59H mutation. The chaperone activity of 6S-NBI-DGJ was also observed on 24 (27%) out of 88 mutated GLB1 enzymes expressed in COS7 cells. This compound significantly reduced the accumulation of GM1 ganglioside in patient fibroblasts, as well as the levels of the autophagy biomarkers p62 and LC3-II. Oral treatment of a p.R201C GM1 gangliosidosis mouse model led to a significant increase in GLB1 activity in heart, liver, kidney, and brain, and to the reduction of GM1 gangliosides and autophagy biomarker (p62 and LC3-II) levels in brain samples. Overall, DGJ-derived compounds have a potential as PC for GLB1 deficiencies, since they increase the enzyme activity of an important number of mutations. Nevertheless, most of the studied MPS IVB mutations seem to be not responsive to these DGJ-derived PC, and a more detailed study of MPS IVB mutations is still required.

Finally, 4-*epi*-IFG-derived compounds have also been studied as PCs for GLB1 deficiencies (Figure 4). In the first case, the compounds (5aS)- and (5aR)-5a-C-pentyl-4-*epi*-IFG were evaluated (Figure 4A) [107]. Inhibition studies using a wild-type human GLB1 showed that (5aR)-5a-C-pentyl-4-*epi*-IFG was about 1600-fold more active than the (5aS)-based compound, with an IC_50_ of 0.008 µM at pH 7.3 and an IC_50_ of 0.180 µM at pH 4.3, suggesting a lower affinity of this compound for GLB1 at the lysosome pH. As observed with other PC for GLB1, (5aR)-5a-C-pentyl-4-*epi*-IFG increased the thermostability of the human wild-type enzyme in a dose-dependent manner. The effect on mutated GLB1 was evaluated in 23 human fibroblasts carrying different missense mutations (Table 2), two of which were associated with an MPS IVB phenotype (p.W273L/p.R482H and p.W273L/p.W509C). An increase in the enzyme activity was observed in 56% of the evaluated mutations, which ranged from 1.5- to 35-fold. Specifically, MPS IVB fibroblasts showed a 1.5-fold increase in the GLB1 activity. Recently, 4-*epi*-IFG-derived compounds carrying methyl, nonyl, and 2-phenylethyl groups at C-5a (Figure 4B–D) were evaluated as potential PCs for human GLB1 [108]. Whereas the 2-phenylethyl- and nonyl-derivates showed IC_50_ similar to or less than that of the previously studied (5aR)-5a-C-pentyl-derived compound, the methyl-derived compound showed a significantly (25-fold) higher IC_50_ than the (5aR)-5a-C-pentyl-derived compound. These results correlated with the enhancement of the enzyme activity in GM1 gangliosidosis patient fibroblasts, with nonyl-derivate showing the most efficient chaperone activity and requiring a 50-fold lower concentration than the (5aR)-5a-C-pentyl- and 2-phenylethyl-derivates to reach the same enzyme activity increase. Nevertheless, nonyl-derivate showed a significantly higher cytotoxicity than the other 4-*epi*-IFG-derived compounds evaluated, which confirmed the potential of (5aR)-5a-C-pentyl-4-*epi*-IFG as a PC for GLB1 deficiency. In fact, the treatment of GM1 gangliosidosis patient fibroblasts with (5aR)-5a-C-pentyl-4-*epi*-IFG restored the maturation of the mutated enzyme and led to the reduction of 31% and 36% of KS and Hex_3_HexNAc_2_ oligosaccharide, respectively.

## 4. Conclusions and Perspectives

During the last years a growing interest has been observed in the identification, characterization, and design of PCs as a treatment alternative for LSDs. In the case of MPS, they have been evaluated only for MPS II, IVA, and IVB, showing these drugs can restore folding and trafficking of the mutated enzyme leading to a recovery of the biological activity of the enzyme. These PCs have been mainly evaluated in vitro using skin fibroblasts for MPS patients, which does not represent the main affected tissues in these disorders. In this sense, it is important to develop cellular models from disease-relevant tissues that allow an effective screening and characterization of PCs for MPS. In addition, the use of virtual screening strategies for the identification of PCs for MPS requires the protein structure of all the lysosomal enzymes involved in GAG catabolism and relevant information on the effect of mutations on protein stability and folding, which will facilitate the identification of PC-responsive mutations. On the other hand, only a couple PCs have been studied in MPS animal models and, more importantly, no clinical trials have been carried out for MPS using this therapeutic strategy. In this sense, it is important to have animal models with missense mutations that allow confirmation of the potential of current and novel PCs for MPS, as well as to accelerate the translation of these results to the clinics. In this last point, drug repurposing may represent a key strategy in identifying novel PCs and in reducing the cost and time of research and development. In addition, it is important to evaluate this therapeutic strategy in other MPS, especially those with CNS involvement, such as MPS III, for which the current therapeutic alternatives and those under development have not shown significant improvement of the CNS impairment.

## Figures and Tables

**Table 1:** Classification of the mucopolysaccharidoses (MPS).

Disorder	Gene	Enzyme Deficiency	OMIM
MPS I (Hurler, Hurler–Scheie, and Scheie syndrome)	*IDUA*	alpha-l-iduronidase	607014 607015 607016
MPS II (Hunter syndrome)	*IDS*	Iduronate-2-sulfatase	309900
MPS IIIA (Sanfilippo syndrome)	*SGSH*	Heparan-N-sulfatase	252900
MPS IIIB (Sanfilippo syndrome)	*NAGLU*	N-acetylglucosaminidase	252920
MPS IIIC (Sanfilippo syndrome)	*HGSNAT*	Acetyl CoA glucosamine N-acetyltransferase	252930
MPS IIID (Sanfilippo syndrome)	*GNS*	N-acetyl-glucosamine-6-sulfatase	252940
MPS IVA (Morquio A syndrome)	*GALNS*	N-acetylgalactosamine-6-sulfate sulfatase	253000
MPS IVB (Morquio B syndrome)	*GLB1*	β-galactosidase	253010
MPS VI (Maroteaux–Lamy syndrome)	*ARSB*	Arylsulfatase B	253200
MPS VII (Sly syndrome)	*GUSB*	β-glucuronidase	253220
MPS IX	*HYAL1*	Hyaluronidase	601492

**Table 2:** Summary of the effect on enzyme activity of pharmacological chaperones for GLB1 deficiencies.

Pharmacological Chaperone	IC_50_/*K_i_* (µM)	GLB1 Mutations	Maximum Activity Enhancement (Fold Change)	Reference
NOEV	IC_50_: 0.2	Patient Fibroblasts p.R201C p.R201H p.R457Q p.W273L * p.Y83H *	5.1 4.5 2.4 2.2 2.0	[102]
Galactose	N.D.	Patient Fibroblasts p.S54N/p.R59C p.R201/p.G579D p.T329A/p.R442Q	1.0 1.4 2.6	[103]
DLHex-DGJ	IC_50_: 6.0 *K_i_*: 0.6	Patient Fibroblasts p.I181K p.R201C p.R201H/p.R457X p.R201H/p.S149F p.R201H/p.H281Y p.R208C/p.W161X p.C230R p.Y270D p.W273L p.A301V p.Y333H p.G438E p.P549L	2.1 9.4 11.1 12.3 12.5 2.5 9.0 1.7 1.3 1.3 1.8 2.3 1.4	[104]
N-(dansylamino)hexylaminocarbonylpentyl-DGJ	*K_i_*: 0.7	Patient Fibroblasts p.R201C	~7.0	[105]
NN-DGJ	IC_50_: 0.12	Patient Fibroblasts p.R351X/p.R351X p.R148S/p.D332N p.R148S/p.R482H p.R201H/IVS14-2A>G p.R201H/p.W509C p.W273L/p.R482H * p.G438E/p.G438E *	1.4 4.1 4.9 7.3 0.9 1.0 1.0	
6S-NBI-DGJ	IC_50_: 32	Patient Fibroblasts p.I51T p.I51T/p.Y316C p.I51T/p.R457Q p.R59H p.G190D p.R201C p.G438E p.R457Q COS7 cells p.I51T p.R148T p.L155R p.G190D p.R201C p.R201H p.R208C p.D214Y p.V216A p.C230Y p.L264S p.N266S p.W273R p.D332N p.K346N p.S434L p.G438E p.Y444C p.R457Q p.R482H p.D491Y p.R590H p.E632G p.D640E	2.6 5.5 4.9 1.1 4.1 4.9 2.7 3.0 1,9 1,8 1,6 1,5 1,9 2,5 1,8 1,4 1,7 2,3 1,9 2,0 1,9 2,2 1,4 2,2 2,2 2,8 2,4 1,5 1,7 2,0 1,6 1,7	[107]
(5aR)-5a-C-pentyl-4-*epi*-IFG	IC_50_: 0.008	Patient Fibroblasts 733 + 2T > C p.I51T p.R59H p.R59H p.C127Y p.R148S/p.S532G/p.L305F p.S191N/R351X p.R201C p.R201C p.R201C/p.H281Y p.R201H/c.247dup1 p.R201H/p.G76E p.R201H/ p.H281Y p.R208C/IVS10 + 1G > A p.Q255H/p.K578R p.H281Y/splicing p.R351Ter/p.R351X p.G438E p.R442Q/p.W92X p.R457Q p.P549L p.W273L/p.R482H * p.W273L/ p.W509C *	1.1 1.6 1.4 1.1 1.1 1.0 11.0 15.0 5.4 18.0 3.5 6.7 4.7 4.4 20.0 35.0 1.0 2.0 1.5 7.3 1.3 1.5 1.5	[107]

N.D.: not determined. * Mutations associated with an MPS IVB phenotype. GLB1 mutations were evaluated in patient fibroblasts or in COS7 cells overexpressing the mutated enzyme
